# Experiences of care for self-harm in the emergency department: comparison of the perspectives of patients, carers and practitioners

**DOI:** 10.1192/bjo.2021.1006

**Published:** 2021-09-22

**Authors:** Sally O'Keeffe, Mimi Suzuki, Mary Ryan, Jennifer Hunter, Rose McCabe

**Affiliations:** School of Health Sciences, City University of London, UK; Unit for Social and Community Psychiatry, Queen Mary University of London, London, UK; London Southbank University, UK; and National Collaborating Centre for Mental Health, Royal College of Psychiatrists, UK; School of Health Sciences, City University of London, UK; School of Health Sciences, City University of London, UK

**Keywords:** Self-harm, suicide, emergency department, liaison psychiatry, qualitative research

## Abstract

**Background:**

Each year, 220 000 episodes of self-harm are managed by emergency departments in England, providing support to people at risk of suicide.

**Aims:**

To explore treatment of self-harm in emergency departments, comparing perspectives of patients, carers and practitioners.

**Method:**

Focus groups and semi-structured interviews with 79 people explored experiences of receiving/delivering care. Participants were patients (7 young people, 12 adults), 8 carers, 15 generalist emergency department practitioners and 37 liaison psychiatry practitioners. Data were analysed using framework analysis.

**Results:**

We identified four themes. One was common across stakeholder groups: (a) the wider system is failing people who self-harm: they often only access crisis support as they are frequently excluded from services, leading to unhelpful cycles of attending the emergency department. Carers felt over-relied upon and ill-equipped to keep the person safe. Three themes reflected different perspectives across stakeholders: (b) practitioners feel powerless and become hardened towards patients, with patients feeling judged for seeking help which exacerbates their distress; (c) patients need a human connection to offer hope when life feels hopeless, yet practitioners underestimate the therapeutic potential of interactions; and (d) practitioners are fearful of blame if someone takes their life: formulaic question-and-answer risk assessments help make staff feel safer but patients feel this is not a valid way of assessing risk or addressing their needs.

**Conclusions:**

Emergency department practitioners should seek to build a human connection and validate patients’ distress, which offers hope when life feels hopeless. Patients consider this a therapeutic intervention in its own right. Investment in self-harm treatment is indicated.

## Background

In the UK, approximately 6000 people take their own life each year.^1^ Self-harm is the strongest risk factor for suicide, defined as intentional self-poisoning or self-injury, irrespective of motive or the extent of suicidal intent.^2^ Self-harm includes acts intended to result in suicide, those without suicidal intent (such as it supports a coping mechanism) and acts where the motivation is mixed or unclear.^3^ For people who self-harm, emergency departments are often the first point of contact with healthcare services: up to 43% of people who take their life attend the emergency department in the year before death.^4^ This makes emergency departments a crucial support system for people in crisis with potential for life-saving interventions. Emergency departments must meet the complex physical and psychiatric needs of people who self-harm, who are known to be at increased risk of suicide.

UK hospitals have sought to meet such complexity through liaison psychiatry services, which are now well established in acute hospitals.^5^ Medical needs are addressed by generalist emergency department practitioners and practitioners from liaison psychiatry teams offer a psychosocial assessment, following National Institute for Health and Care Excellence (NICE) guidelines.^6^

## Aims

Previous research has explored staff views on different kinds of liaison services in integrating physical and mental healthcare,^7^ along with patients’,^8,9^ young people^10^ and carers’^11^perspectives of care for self-harm in the emergency department. The aim of this study was to compare and integrate the perspectives of generalist emergency department practitioners, liaison practitioners, adult patients, young people and carers on delivering and receiving care for self-harm in emergency departments in England.

## Method

### Setting

This study is part of the ‘Improving outcomes in patients who self-harm – Adapting and evaluating a brief pSychological inteRvention in Emergency Departments’ (ASsuRED) study. The overall aim of the ASsuRED study is to adapt and test an intervention for people presenting to emergency departments with self-harm in England (www.assuredstudy.co.uk). The COREQ-checklist, a guideline for reporting qualitative research, was used in reporting this study (see Supplementary Data 1 available at https://doi.org/10.1192/bjo.2021.1006).^12^

### Participants

To explore perspectives and experiences of delivering care in England, we recruited both generalist emergency department and liaison psychiatry practitioners. Practitioners were recruited from four emergency department and liaison psychiatry teams across London and the South West of England. An email from the team manager was circulated inviting them to a focus group and those who wished to take part attended. We sought perspectives from a diverse range of practitioners, including doctors, nurses and psychologists.

To explore experiences of receiving care, we recruited people with experience of attending emergency department as patients or carers. We use the term carer broadly – a trusted other who has attended the emergency department in a supportive capacity (usually a family member/friend). Patients and carers were recruited through mental health charities, service user groups (including the McPin Foundation), the National Self Harm Network and the Service User and Carer Group Advising on Research at City University of London. An advert was circulated among these groups and posted on social media. Those interested in taking part contacted the research team. We sought diversity in gender, age (including young people aged 16–25 years), ethnicity and first versus multiple emergency department attendances; and sought to include carers with a range of relationships with patients (parents, spouses, friends). Patients and carers were offered a £15 voucher for participating.

### Data collection

Data were collected in focus groups and individual interviews between September and December 2019. Data collection took place in meeting rooms in hospitals (with staff) and on university premises (with young people, patients and carers). Whenever possible, focus groups were used to facilitate exchange of views and allow participants to build on each other's perspectives. When participants could not attend or were uncomfortable in a group, individual interviews were conducted. Focus groups and interviews were conducted in person and facilitated by postdoctoral (S.O.’K. and J.H.) and postgraduate (M.S.) researchers, using a semi-structured topic guide developed with a Lived Experience Advisory Panel (LEAP), exploring experiences of delivering/receiving care. Open questions were used and the topic guide was used flexibly, allowing the conversation to be focused on the issues most salient to participants (see topic guide in Supplementary Appendix 1). A member of the LEAP (M.R.) was involved in data analysis and co-authored this manuscript.

Eleven focus groups and 14 interviews were conducted. These were audio/video-recorded, according to preference. Focus groups lasted 28–102 min, average 65 min, and interviews lasted 28–67 min, average 48 min. Data were transcribed verbatim, anonymised and checked by the researchers for accuracy.

### Data analysis

Data were analysed using framework analysis to facilitate comparison of different stakeholder perspectives in a complex data-set by a research team.^13^ Data were organised using NVivo Version 12.0.^14^ Framework analysis comprised five stages: familiarisation with the data, identifying a framework, indexing, charting and mapping, and interpretation. Familiarisation involved listening to and reading the transcripts. We then identified a framework of categories to organise the data broadly based on *a priori* topics. Indexing involved coding each part of the transcript into the relevant category. Coded data were then charted, whereby the raw text was summarised into the framework matrix. Once complete, this provided a manageable format to proceed to ‘mapping and interpretation’.

To interpret the data, for each stakeholder group (young people, adults, carers, liaison practitioners, emergency department practitioners), the framework matrix was interrogated to identify patterns relating to their experiences of receiving/delivering care. This was carried out independently by S.O.’K., M.S. and J.H., who then came together to compare and integrate their interpretations. The objective was to reach consensus on the themes that best depicted the complexity of the data. Where there were disagreements in our interpretation of the data, we returned to the raw data and discussed it until we reached agreement. This team approach enabled us to reflect on our preconceptions and biases throughout the analysis. After agreeing themes within each stakeholder group, we compared the themes across stakeholder groups to integrate them to produce a shared narrative incorporating the perspectives of all stakeholder groups.

### Ethical considerations

The authors assert that all procedures contributing to this work comply with the ethical standards of the relevant national and institutional committees on human experimentation and with the Helsinki Declaration of 1975, as revised in 2008. All procedures involving human patients were approved by London-Surrey Borders Research Ethics Committee (Reference: 19/LO/0778). Written informed consent was obtained from all participants. In order to protect the confidentiality of participants, identifiable information has been removed.

## Results

Nineteen people with experience of attending the emergency department for self-harm – 7 young people (under 25 years) and 12 adults (over 25 years), 8 carers, 15 generalist emergency department practitioners and 37 liaison psychiatry practitioners participated. Participant demographics are shown in Table 1.

**Table 1:** Demographic characteristics of participants

Characteristic	Patient (*n* = 19)	Carers (*n* = 8)	Practitioners	
			Generalist emergency department (*n* = 15)	Liaison psychiatry (*n* = 37)
Age, years: mean (range)	39 (17–77)	59 (48–77)	39 (22–60)	37 (21–63)
Gender, *n* (%)				
Female	16 (84)	8 (100)	6 (40)	27 (73)
Male	3 (16)	–	9 (60)	10 (27)
Ethnicity, *n* (%)				
White British	8 (42)	4 (50)	7 (47)	13 (35)
White other	3 (16)	–	2 (13)	6 (16)
Asian	4 (21)	–	3 (20)	10 (27)
African	1 (5)	–	–	2 (5)
Caribbean	–	2 (25)	–	–
Black/Black British	–	–	–	5 (14)
Other ethnic group	2 (11)	2 (25)	1 (7)	1 (3)
Missing	1 (5)	–	2 (13)	–
Carers				
Spouse	–	2 (25)	–	–
Friend	–	2 (25)	–	–
Child	–	6 (75)	–	–
Profession, *n* (%)				
Consultants	–	–	2 (13)	–
Psychiatrists	–	–	–	4 (11)
Junior doctors	–	–	9 (60)	11 (30)
Nurses	–	–	4 (27)	18 (49)
Psychologists	–	–	–	3 (8)
Occupational therapists	–	–	–	1 (3)

We identified four themes. All stakeholders agreed the following theme.

1. The wider system is failing people who self-harm: they can only access crisis support as they are often excluded from services, leading to unhelpful cycles of attending the emergency department.

Stakeholders held different perspectives on the following three themes.

1. Practitioners feel powerless and become hardened towards patients, with patients feeling judged for seeking help which exacerbates their distress.
2. Patients need a human connection to offer hope when life feels hopeless, yet practitioners underestimate the therapeutic potential of interactions.
3. Practitioners are fearful of blame if someone takes their life: formulaic question-and-answer risk assessments help make staff feel safer but patients feel this is not a valid way of assessing risk or addressing their needs.

### Theme 1: the wider system is failing people who self-harm: they can only access crisis support as they are often excluded from services, leading to unhelpful cycles of attending the emergency department

All stakeholders agreed that the wider system is failing people presenting with self-harm. They described an inadequate healthcare system which excludes many people from treatment. People described not being able to get a general practitioner appointment, long waiting lists and narrow referral criteria for services that often exclude people with self-harm and those who have complex social, psychological and emotional needs (Appendix 1, quote 1). For instance, people without a diagnosable mental health disorder would not meet criteria for secondary mental health services (Appendix 1, quote 6). An inadequate care pathway for people who self-harm led to lack of continuity of care and poor communication between services.

Patients described having no support other than crisis care. For some this meant they were sign-posted back and forth between the emergency department and crisis team, with nothing in place in the community to avoid reaching crisis point (Appendix 1, quote 2). Practitioners described doing their best to contain the crisis in the short-term but paucity of community mental health and voluntary sector services limited the referrals emergency department practitioners could make. People reported coming back to the emergency department repeatedly in crisis because of the lack of support in the community. This added to the burden on stretched emergency departments in which practitioners were fire-fighting, focused on risk and unable to address the issues underlying self-harm (Appendix 1, quote 5).

Practitioners were often heavily reliant on people's own support network. People attending with a carer were considered as having social support and thus lower risk, so were more likely to be discharged from the emergency department. This was difficult for carers, who described feeling underinvolved in decision-making in the emergency department, and then over-relied upon and often ill-equipped to keep the person safe (Appendix 1, quote 4). Carers emphasised the need for support for carers, as well as greater support for the patients themselves.

All stakeholders described that people needed more than a crisis-only response to stay safe in the longer term (Appendix 1, quote 3). Positive experiences were when individuals were being provided with good follow-up care. One young person had an immediate referral to the children and adolescent mental health services team, providing ongoing support. However, for many patients, appropriate services were not available to provide follow-up care, leaving practitioners frustrated with not being able to offer more to people, because of a fragmented and disjointed healthcare system.

### Theme 2: practitioners feel powerless and become hardened towards patients, with patients feeling judged for seeking help which exacerbates their distress

Both young people and adult patients reported feeling shame and guilt for seeking help in the emergency department for self-harm (Appendix 2, quote 7). This was exacerbated by difficult interactions with practitioners, linked to practitioners’ feelings of being powerless, burntout and becoming less responsive towards patients’ distress.

The comparison between mental and physical health came up repeatedly. People felt like ‘time wasters’, that they were using resources unnecessarily or less worthy than those with physical health issues, made worse by the chaotic environment and long waiting times in the emergency department. The stigma felt by patients was striking, and similarly those carers who were parents described feeling that both they and their child were being judged, such as one carer who described feeling like a ‘bad mother’. Patients and carers emphasised the need for non-judgemental treatment (Appendix 1, quote 8). People with positive experiences of care were those who felt validated by practitioners, in contrast to difficult experiences for those who felt self-harm was not perceived as a legitimate reason to attend the emergency department.

For those with negative experiences, feelings of guilt and worthlessness were exacerbated by practitioners’ responses, when their distress was not taken seriously. For example, one person described how a practitioner said: ‘We're not going to make it too comfortable for you to come here or we're enabling you’. Being discouraged from attending came up repeatedly, including from generalist emergency department practitioners concerned that making the emergency department environment nicer might encourage people to attend more. Generalist emergency department practitioners also described that people with self-harm would not automatically be referred to the liaison psychiatry team, which is contrary to NICE guidelines.

Difficult experiences were prominent for people with a diagnosis of borderline personality disorder and those labelled as ‘frequent flyers’. Such labels had a negative impact on the way some practitioners treated people. One young person overheard nurses describe her as ‘the attention-seeking type, the dramatic type, the crazy one’. This was detrimental for this person, who already felt vulnerable.

Practitioners wanted to help but felt powerless. They recognised complex, long-standing problems but did not believe they could meet someone's needs or help them to stop self-harming. Negative attitudes when people re-attend were linked to powerlessness and frustration. Experience of burnout was described (Appendix 2, quote 9) as becoming ‘hardened’ or ‘cold’ towards patients – which may in turn exacerbate feelings of worthlessness by patients.

### Theme 3: patients need a human connection to offer hope when life feels hopeless, yet practitioners underestimate the therapeutic potential of interactions

Patients strongly felt the most important thing was a human connection with the liaison practitioner, to offer hope at a time when they felt hopeless. People described how a meaningful interaction would instil hope in the person and could make them safer when leaving hospital. Patients and carers described how important it was to feel heard and for practitioners to show empathy, compassion and reassure them it was right to seek help. Good experiences were when the practitioner was ‘not intimidating’ and explained what they were doing and why. People did not expect the practitioner to problem solve or focus on the positives – they simply needed to be listened to and understood (Appendix 3, quote 10). The importance of building rapport and trust was emphasised (Appendix 3, quote 11). Open conversations through a human connection improved the validity of the information shared by the patient – allowing practitioners to get a more accurate picture of risk and better understanding of their needs, so that they could provide more useful recommendations and develop personalised safety plans. These views were shared among young people, adults and carers alike.

Generalist emergency department practitioners recognised the therapeutic value of the person feeling listened to, but felt the emergency department was not the right environment for therapeutic conversations (Appendix 3, quote 12). Generalist emergency department practitioners felt limited in their ability to build a human connection with the person.

Liaison practitioners did not see their role as to ‘treat’ or ‘offer intervention’ to patients, but to manage their short-term safety with any potential therapeutic value a bonus, rather than a core aim, of assessment. One practitioner acknowledged that the therapeutic value of these interactions was easily overlooked (Appendix 3, quote 13). As practitioners will often not see the person again, they sometimes undervalued the impact of a compassionate interaction. For patients these connections – even brief, one-off interactions – could make a difference and instil hope. This emphasis on human connection was strongly linked with the next theme, where practitioners described barriers in forming human connections with patients.

### Theme 4: practitioners are fearful of blame if someone takes their life: formulaic question-and-answer risk assessments help make staff feel safer but patients feel this is not a valid way of assessing risk or addressing their needs

Practitioners strongly emphasised that their role was to manage risk. Patients and carers perceived this focus on risk as making interactions with practitioners procedural and superficial.

Practitioners spoke extensively about the multiple layers of risk they were managing: risk to themselves, patient risk and departmental risk: ‘with the work we do, our head is always thinking its risk, risk, risk. When you think of risk how do you mitigate those risks, that's the way we think’. Practitioners were fearful of being blamed, feeling responsible for identifying risk and keeping someone safe: ‘The thought of a life on your hands for the rest of your life is really hard … that's a big burden for people to carry’ (generalist emergency department practitioner).

Practitioners described the ‘witch-hunt’ that would ensue if someone did end their life, and fear of being in the coroner's court. This weighed heavily on the minds of practitioners. Risk assessments were used to protect the practitioners and the organisation – which led to detailed documentation. Practitioners described typically spending twice as long documenting an assessment as the time spent with the patient. Patients perceived the paperwork being done for the organisation, rather than because it was helpful for the person in crisis. Practitioners assessed risk in a formulaic question-and-answer assessment for the purpose of the records, which patients perceived as a superficial interaction, failing to get to the ‘root cause’ of their self-harm (Appendix 4, quote 16).

Patients felt that practitioners ‘cover their backs’ and carers shared the view that risk assessments felt like a ‘tick-box’ exercise. One person described feeling she was ‘talked into’ downplaying her risk by practitioners (Appendix 4, quote 14). One practitioner described the narrow way in which risk was often viewed in mental health services – differing from risks from the patient's perspective (Appendix 4, quote 17). Patients described how difficult it was to speak honestly to a practitioner when in crisis, needing to feel safe to share innermost feelings (Appendix 4, quote 15).

## Discussion

### Main findings

There were two key findings from this study. First, young people, adults, carers and practitioners in the emergency department agreed that the wider healthcare system was failing and excluding many people who harm themselves. As a result, they presented in crisis to the emergency department, often repeatedly. Second, the quality of psychosocial assessment could be improved. The current focus on formulaic risk assessment, driven by practitioners’ fear of being blamed if someone takes their life, was an obstacle to a therapeutic assessment. A human connection was valued most highly by patients and instils genuine hope when many feel their life is not worth living – a view that was emphasised by young people, adults and carers.

### Strengths and limitations

Strengths of this study were the relatively large sample size and integration of perspectives of generalist and mental health practitioners, patients and carers, providing a rich insight into emergency department treatment for self-harm. Interviews and focus groups used open questions, so responses were largely spontaneous and findings were grounded in participants’ experiences. A person with lived experience was part of the research team and contributed to the study design and analysis.

We acknowledge limitations of the sampling approach. Patients and carers proactively responded to leaflets, flyers or social media posts inviting participation. Those who opted to take part in the study may have done so because of having particularly negative experiences. In contrast, practitioners who participated may be those with greater interest in mental health and self-harm. We obtained a reasonable proportion of participants from minority ethnic backgrounds, but acknowledge that males were underrepresented among participants with lived experience, especially among young people.

### Comparison with existing research

A 2008 systematic review of studies published between 1973 and 2007 reported on people's experiences of hospital treatment for self-harm.^15^ The present study suggested many of the issues experienced by people have not changed over this time period, with people continuing to feel misunderstood and self-harm being perceived as an illegitimate reason for attending the emergency department.^15^ Consistent with recent findings, this study showed how compassionate care can foster a therapeutic interaction, whereas assessments that are perceived as generic, formulaic and uncaring were considered unhelpful and resulted in iatrogenic harm for some people.^9,16^ This fits with recent findings from the perspective of young people in Australia, who emphasised how emergency department care was countertherapeutic.^10^ In line with previous findings about what matters from the perspective of young people and adults seeking help, we found that helpful treatment in the emergency department is being respected, believed and taken seriously.^10,17^ The therapeutic value of having someone to talk to was emphasised by patients and carers alike – which is essential for people to fully disclose their experiences to practitioners and for practitioners to conduct a valid risk assessment.^18^ A human connection was considered most important for patients, where a therapeutic encounter could instil hope at a time when life does not feel worth living, and can potentially be life-saving, as reported in previous studies.^8,16^ This demonstrates the importance of building a therapeutic alliance with patients, consistent with randomised controlled trials of interventions demonstrating a link between a stronger therapeutic alliance and fewer suicide attempts.^19^ This indicates potential for such interventions that could be delivered in the emergency department context.

Our findings were similar to those from a systematic review published a decade ago, which found that hospital staff generally had negative attitudes and feelings of frustration towards patients who self-harm.^20^ Emergency department care has previously been described as hostile, with those with histories of trauma or a diagnosis of personality disorder describing particularly difficult and stigmatising experiences,^9,21^ overlapping with the experiences of many patients and carers in the present study. There was stigma associated with seeking help for mental health problems, compared with physical health problems, with people feeling less worthy of treatment. The sense of stigma experienced by people was striking, which was of significant concern as such experiences could discourage future help-seeking.

In this study, stigmatising behaviour from staff appeared to be partly a result of practitioners feeling demoralised and powerless as they were repeatedly seeing patients failed by the mental health system. As there was little ongoing support and treatment, people mainly sought help when in crisis with some people attending the emergency department multiple times a year. Emergency department practitioners felt frustrated by patients re-attending, powerless to help them and over time found it hard to feel empathy. This was not surprising given that emergency departments are penalised financially (with fines) when people attend over a certain number of times in a year. The issue of burnout in the emergency department context was raised by practitioners, who were at risk of burnout because of exposure to distressed individuals, pressure to discharge people within a set time frame and little continuity with patients after discharge.^22^ This was coupled with little support and supervision for emergency department practitioners.

Recent research showed that risk assessment can de-humanise the clinical encounter.^16,23^ Our findings corroborate this, as patients often perceived interactions with practitioners as superficial and that practitioners were ‘box-ticking’ to ‘cover their backs’. In this study, practitioners spoke of the extensive documentation required for each assessment they conducted, often spending twice as long documenting an assessment than time spent with the person, in line with findings from previous studies.^5,7^ Mental health record systems have been described as being unfit for purpose for high-volume, low-contact services such as the emergency department, compared with mental health services who have smaller case-loads and ongoing patient contact.^7^ This in part explained the formulaic question-and-answer style assessments that were perceived as superficial by patients. This indicated that a better balance between organisational and patient priorities is needed.

Findings from this study were consistent with recent evidence that the needs of people who harm themselves are not being met, as they face significant barriers to accessing support in the community.^24^ The emergency department is considered the wrong place at the wrong time for many and is a last resort for people who cannot access help elsewhere.^25^ The role of liaison psychiatry includes offering sign-posting, referrals and treatment in the community for people presenting to the emergency department with psychiatric needs^26^– yet the effectiveness with which they could do this was severely limited by lack of available services.

Practitioners acknowledged the expectation to discharge patients even when they recognised the person may not be safe and their over-reliance on carers to keep the person safe after discharge. Failure to receive appropriate and timely support in the community often led to the revolving door of the person repeatedly coming to the emergency department in crisis, without support to prevent them from reaching crisis point. This echoes findings from a Samaritans report, which described how people are ‘pushed from pillar to post’ between services.^24^

Evidence has indicated that a psychosocial assessment after self-harm reduced the risk of repeat self-harm.^27^ NICE guidelines state all people who present with self-harm should receive a psychosocial assessment,^2^ yet emergency department practitioners in this study reported that those who self-harm would not necessarily be referred to liaison psychiatry, based on their understanding of NICE guidelines. This may in part explain why only 60% of patients attending hospital for self-harm receive a psychosocial assessment.^28^ In response to this issue, a national Commissioning for Quality and Innovation (CQUIN) was implemented in 2020–2021 that aimed to increase the number of patients presenting to hospital with self-harm who receive psychosocial assessments. This CQUIN should go some way to improving adherence to NICE guidelines for self-harm in emergency departments, although to date the effectiveness of its implementation has not been reported.

### Recommendations

Organisational requirements should be there to improve patient care – yet current systems emphasise assessing risk and documenting this to protect the organisation, which, for patients and carers, does not optimise the opportunity for a therapeutic interaction to reduce the person's distress. Shifting from the current model of risk assessment to a more therapeutic approach to risk assessment requires cultural change within organisations, to support practitioners to conduct less formulaic and more person-centred assessments.

A cultural shift is needed away from the ‘witch-hunt’ if a patient takes their life, to developing postvention responses to support practitioners.^29^ For instance, some National Health Service (NHS) trusts have changed policy so that the coroner's courts would be attended by senior management rather than the responsibility being on individual clinicians.

Experiences of staff burnout were reported by practitioners in this study. Training for staff is needed to overcome stigmatizing attitudes towards self-harm, as research has found that education had positive effects on staff attitudes towards self-harm.^30^ To date there is no standard model of staff training for those regularly treating self-harm^20^ yet this would be a positive step towards challenging stigmatising attitudes that continue to be experienced by patients – particularly for nurse practitioners who assess the majority of patients presenting to the emergency department with self-harm. Regular supervision for staff is indicated, as practitioner well-being is associated with patient satisfaction and safety^22^ and evidence suggests that supervision is associated with greater job satisfaction and lower levels of stress.^31^

Research is needed to develop the evidence base for such interventions delivered in the emergency department, such as the Attempted Suicide Short Intervention Program, that emphasises how building a human connection can give patients hope at a time when life does not feel worth living.^32^ A randomised controlled trial of this approach found that the quality of the therapeutic alliance was associated with fewer repeat suicide attempts after 24 months,^19^ supporting patients’ reports that forming a human connection in times of crisis is therapeutic in itself.

In the UK context, the NHS Community Mental Health Framework has been developed to support the NHS Long Term Plan, for a whole-system, whole-person approach to care within primary care and the voluntary, community and social enterprise sector. With significant investment, a principal aim is to provide care and support for many people whose needs are not being met in the community. Evaluation of the Community Mental Health Framework should assess improvements in care for people who self-harm, who have been deprived of timely access to treatment for many years, as described by young people, adults and carers in the present study.

Notwithstanding the lack of pathway and community services in the healthcare system, these findings have implications for how practitioners can improve patient care within existing resources in the following ways.

- Focus on building a human connection with patients. A therapeutic interaction can provide hope to patients when they feel their life is worthless and reduce their distress, thus making them feel safer when leaving the emergency department.
- If patients feel safer leaving the emergency department, this will decrease burden on carers.
- Validate distress. This helps to establish trust and promote disclosure, which ultimately will lead to a more valid assessment of risk and will allow practitioners to provide advice that is more tailored to the person's needs.

## Data Availability

The data that support the findings of this study are available from the corresponding author (S.O.’K.), upon reasonable request. The data are not publicly available as they contain information that could compromise the privacy of research participants.

## Supplementary material

For supplementary material accompanying this paper visit http://dx.doi.org/10.1192/bjo.2021.1006. click here to view supplementary material

## Appendix 1

Quotes for each theme from each stakeholder group for Theme 1

**Table:**

Theme 1: the wider system is failing people who self-harm: they can only access crisis support as they are often excluded from services, leading to unhelpful cycles of attending the emergency department	
Patient (41-year-old woman) Patient (35-year-old woman) Patient (31-year-old man)	Quote 1: ‘Many of us can't get support from mental health services. We're kind of excluded so there's no one to liaise with. We're not getting anything, except a borderline personality diagnosis.’ Quote 2: ‘They'll say well, I'm sorry, there's no beds but maybe you could go home and wait and work with the crisis team and that is the circle, so now I don't go until I am literally at death's door, or I've done something and usually when you've done something they'll say well why didn't you call us, and it's like well what's the point and I find that with the crisis team, they'll say “if you're thinking of doing this, take yourself to A&E” [accident and emergency]. A&E's like “have you done anything, no, go the crisis team”, you know, erm, and I just don't think the crisis team is enough to contain you when you are in, at that point where you think if I go home I, I'm so scared and so anxious I'm going to do something to myself.’ Quote 3: ‘Safety planning could be how do you keep yourself safe and look after yourself. Not necessarily in moments of crisis, but all the time. How can we put a plan in place where, you know you can maybe follow these steps and do these things that can help you get out of this situation? Not, you know, who do you call when you're on the train platform or something.’
Carer (55-year-old mother attending emergency department with daughter)	Quote 4: ‘Sometimes when she was being assessed by psych liaison, they wanted her to be discharged under my care, but without me being involved in this conversation. I thought “what the heck are you talking about?”. I mean the fact that they were making a decision but not involving me was one thing, but [my daughter] was adamant that I am not her carer, I am not a professional, that is totally not acceptable for her. They're saying yes we agree you're not safe to be left alone, but we don't have anywhere else for you to go, so you have to go home.’
Practitioner (generalist emergency department)	Quote 5: ‘There's a system lots and lots of risk assessments and analysis of what the problem is but never actually getting far enough down the line with each individual to actually provide the therapeutic benefit. The whole system is in so much crisis then they end up [in the emergency department], everything gets front loaded because there's so many people and so everything's focused on a risk assessment and we haven't got the capacity to actually treat those people which means they are more likely to come back in another crisis.’
Practitioner (liaison psychiatry)	Quote 6: ‘For people that are presenting with self-harm, who don't have a necessarily diagnosable mental disorder, they therefore have no access to a service. But everybody else isn't sufficiently skilled to manage their risk, or I mean there really is very little for those people it feels. They sit in that kind of middle of the gaps.’

## Appendix 2

Quotes for each theme from each stakeholder group for Theme 2

**Table:**

Theme 2: practitioners feel powerless and become hardened towards patients, with patients feeling judged for seeking help which exacerbates their distress	
Patient (21-year-old woman)	Quote 7: ‘I'm here because I've almost put myself here, when there could be someone who's having a heart attack or has done something not, and they just, and you're like, I, I feel bad, because I feel like I'm taking up their time.’
Carer (77-year-old carer attending emergency department with son)	Quote 8: ‘They just think that it's self-inflected. You come here, you're wasting our time, let's get you patched up and we can get on with our business. They need to have somebody that's not judgemental, that's going to look at them, and see them as a person, not as somebody that's taking their time up.’
Practitioner (generalist emergency department)	Quote 9: ‘The environment that we're in when you start burning out, so one of the first signs of burning out is not really caring about your patients anymore, so that's the danger here, ’cause when you really start getting knackered you just don't care, you just get fed up with people rocking up time and time again self-harming, telling myself y'know it'd be a lie to say you haven't done it, you just think its f----g them again y'know, I think it does happen I mean we'd be crazy to say it doesn't because it does.’

## Appendix 3

Quotes for each theme from each stakeholder group for Theme 3

**Table:**

Theme 3: patients need a human connection to offer hope when life feels hopeless, yet practitioners underestimate the therapeutic potential of interactions	
Patient (57-year-old man)	Quote 10: ‘You want to be heard, you want to be seen. I want to be seen, I want to be hearing something with a bit of depth rather than the superficial things that you're trying to tell me to, that doesn't do me anything.’
Carer (65-year-old mother)	Quote 11: ‘You have to build up rapport, you have to build up a relationship for them to open up and talk. If you go jumping right in they're thinking you're in their personal space and they're not ready to talk. So, you know, just feel it out and try to get the understanding of the person, where they're coming from. You have to have this persona about you that you're there to support. You're not there to penalise or to embark on their personal space.’
Practitioner (generalist emergency department)	Quote 12: ‘I don't wanna then start a situation where I'm asking questions that they're gonna be asking half an hour later which in a way makes people quite upset and angry because it feels like they're just being asked the same questions over and over again.’
Practitioner (liaison psychiatry)	Quote 13: ‘I guess people mentioned that we talk about the function of the assessment. I think we always think that the risk assessments are kind of the main thing. And it is the main thing, but then we perhaps undervalue or we don't realise how therapeutically beneficial they can be. Um and we, it's easy to remember with the people that we see 12 hours later [but], we forget all the people that we see. We do a risk assessment, but actually the interview itself is quite therapeutic and beneficial, and we tend to forget those because we don't see them again.’

## Appendix 4

Quotes for each theme from each stakeholder group for Theme 4

**Table:**

Theme 4: practitioners are fearful of blame if someone takes their life: formulaic question-and-answer risk assessments help make staff feel safer but patients feel this is not a valid way of assessing risk or addressing their needs	
Patient (29-year-old woman) Patient (20-year-old woman)	Quote 14: ‘I've almost felt um, manipulating is far too strong a word, but almost manipulated into agreeing that my risk is lower, because it felt like a tick-box exercise. So like I've had people sort of phrase questions like, “oh, you don't need any extra support, do you?” And it's almost felt like these really leading questions, um, and then questions from one practitioner, when I'd said that I'd felt suicidal on and off since I was about 12, asking, well why haven't you done it yet then? And like I know that that question can be phrased in a different way, that's strengths based, and that's useful, but the sort of phrasing and the tone came across very like, well you're obviously not risk, tick, sort of thing. And I think yeah, that obsession with … it doesn't even feel like risk assessment, it feels like have we covered our back?’ Quote 15: ‘But actually, you know, it should be like an environment where like being open and honest in that way is kind of “praised”. Like as in praised with kind of, you know, a proper response and listening and talking, and not, not like then passing onto someone else, or disengaging and saying like oh you're too much of a risk or whatever.’
Carer (56-year-old mother)	Quote 16: ‘People really are not robots. When they come to that place where they want to self-harm or suicide, there has got to be a root cause and the initial thing should be, not to assess like oh you are some kind of robot, oh look he's talking to himself, he's twitching, he's been taking drugs. No. Why. The question why. You know, like you say talk to me. Like someone would say “talk to me, tell me something, why are you at this desperate state, talk, tell me something”. So, I think that is good, that is very good. Because there is always a root, there is always a root cause to something.’
Practitioner (liaison psychiatry)	Quote 17: ‘It's amazing how in, in mental health we get so narrow on risk to self or others and I think a lot of that is anxiety about we will be held responsible if anything happens. But they're not, we're, from our client's point of mind they're not the risks that are coming up in their minds. The risks are all this other stuff that happens in people's lives which are in some ways much more important, so I think yeah, if we're thinking about, about safety planning, okay if it's people presenting with self-harm we want them to have other ways of coping other than harming themselves but that maybe not be the only bits that are important in terms of their safety. Often what we think of as important isn't. You know, why is this woman drinking out on the street? Okay, it's because if she drinks in her home she gets sexually abused.’
