# Supplementary material for: Experiences of care for self-harm in the emergency department: comparison of the perspectives of patients, carers and practitioners
Source: BJPsych Open. 2021 Sep 22;7(5):e175. doi: 10.1192/bjo.2021.1006 (PMC8485342; doi:10.1192/bjo.2021.1006)
Supplement: Supplementary file 1 [file bjosup.zip › S2056472421010061sup001.docx]

**Supplementary Appendix 1. Topic guide**

The following questions and prompts serve as a guide for the focus groups and interviews.

**Patients / Carers**

- What is your experience of current practice in Emergency Departments for people presenting with self-harm?
- What would be helpful for patients presenting to Emergency Departments?
- What is unhelpful for patients presenting to Emergency Departments with self-harm?
- What is missing from current standards of care for people who self-harm?

**Practitioners**

- What do you think about current practice in ED for people presenting with self-harm / suicidal ideation?
- What works well when supporting people who have self-harmed?
- What concerns do you have when working with people who have self-harmed?
- What goes through your head when people come in with self-harm? And how do you feel when patients reattend?
- What is missing from current standards of care for people who self-harm?
